# snz/SNX25 at the crossroad of endocytosis and lipid handling in autophagy

**DOI:** 10.1080/27694127.2022.2047267

**Published:** 2022-03-22

**Authors:** Annie Lauzier, Steve Jean

**Affiliations:** médecine et des sciences de la santé, Département d’immunologie et de biologie cellulaire Université de Sherbrooke Rue Jean-Mignault Sherbrooke, Québec, 3201, Canada, J1E 4K8

**Keywords:** Autophagy, lipid metabolism, snazarus, sorting nexin 25, VAMP8

## Abstract

Macroautophagy/autophagy is coupled to a myriad of intracellular processes, among which vesicular trafficking is an important contributor of membranes and proteins required at various stages, from autophagosome formation to degradation. Hence, understanding how membrane trafficking is coupled to autophagy induction and how cells rewire trafficking upon high autophagic needs is instrumental to our understanding of autophagy. In our recent manuscript, we tested a known class of endosomal sorting regulators, the sorting nexin family, for their involvement in autophagy in *Drosophila*. We identified *snz* (snazarus) as an important regulator of autophagy in *Drosophila* as well as in mammalian cells, by demonstrating a role for the *snz* human ortholog *SNX25* in HeLa cells. Using knockout rescue experiments, we observed that *SNX25* loss affects many cellular processes, namely VAMP8 endocytosis and lipid handling. Mutational studies identified separate protein domains involved in these processes. Given the role of this sorting nexin family in lipid droplet regulation, our work expands their requirement to autophagy.

Autophagy relies on membranes and lipid influxes from a variety of organelles to feed phagophore expansion, leading to autophagosome formation. The phagophore and autophagosome lipid composition is also dynamically regulated during growth, maturation, and autophagosome-lysosome fusion. In addition to phagophores and autophagosomes, other vesicular compartments must account for and adapt to variations in autophagic needs. As such, vesicular trafficking of autophagic regulators must be tuned to demand, and mechanisms regulating this coupling are just being uncovered.

An important class of vesicular trafficking regulators is the sorting nexin (SNX) family. Through the phox homology (PX) domain, SNXs bind various phosphoinositide species, allowing their recruitment to intracellular membranes where they modulate endocytic and sorting events. Given their central roles in trafficking, different members affect autophagosome generation, but very few were identified as regulating terminal steps of autophagy. To test for SNX involvement in late stages of autophagy, we screened all *Drosophila* SNXs for their requirement in autolysosome formation [1]. From this focused RNAi screen, we identified the gene *snz* (snazarus) as an important regulator of autolysosome formation. Indeed, decreased expression of *snz* by RNAi or *snz* deletion in flies leads to ref(2)P and autophagosome accumulation. Together, these results demonstrate defective autophagy and decreased autophagosome clearance upon snz loss.

We then tested the four *snz* human paralogs, namely SNX13, SNX14, SNX19 and SNX25, in HeLa cells. We found that *SNX14* and *SNX25* loss, through RNAi or CRISPR-induced gene inactivation, result in autophagosome accumulation, indicating functional conservation. Because autophagosome buildup can be caused by defective endolysosomal compartments, we assessed endolysosome functions and overall endosome distribution in flies and HeLa cells upon *snz* or *SNX25* loss, respectively. Endolysosomes are mostly unaffected, except for a slight increase in lysosome numbers in *snz*-depleted flies. This led us to test the localization of the *Drosophila* R-SNARE Vamp7 in flies and its ortholog VAMP8 in mammals, because Vamp7/VAMP8 trafficking is modulated by starvation and is important for autophagosome-lysosome fusion. Interestingly, we observed Vamp7/VAMP8 accumulation close to, or at, the plasma membrane in *snz*/*SNX25*-depleted cells and detected proximity between snz-Vamp7 and SNX25-VAMP8, suggesting a functional link.

The multi-domain-containing protein snz and its human orthologs (Fig. 1A) display specific functions linked to lipid droplet formation and lipid stress handling, which are conveyed through independent domains. To test a role for the individual domains, we performed rescue experiments by expressing *SNX25* deletion mutants in *SNX25* KO HeLa cells and monitored VAMP8 internalization. We focused on VAMP8, given its plasma membrane accumulation and reduced internalization in *SNX25* KO cells. Surprisingly, the SNX25 TM domains are dispensable for VAMP8 internalization, whereas the PX domain is required. Mammalian SNX13, SNX14, and SNX25 PX domains have distinct preferences for phosphoinositides, with the snz PX domain more closely resembling the SNX25 PX domain. Considering the VAMP8 uptake defect upon *SNX25* deletion, the SNX25 PX domain requirement for VAMP8 internalization and the preferred SNX25 PX domain interaction with di-phosphorylated phosphoinositides, we hypothesized that SNX25 can potentially affect endocytosis more generally. This hypothesis was not corroborated, and no effects are observed on clathrin-dependent and -independent endocytosis upon SNX25 loss.

**Figure 1.**
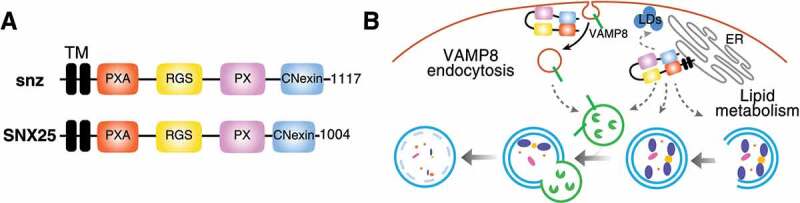
Potential roles of snz and SNX25 in autophagy. (**A**) Domain organization of snz and SNX25. (**B**) snz/SNX25 are required for proper Vamp7/VAMP8 internalization independently of endoplasmic reticulum (ER) localization (left part of the model). snz/SNX25 can potentially affect lipid exchange between the ER and endolysosomes or autophagosomes. snz/SNX25 can also modulate lipid droplet biogenesis, potentially affecting autophagy (right part of the panel).

Although VAMP8 uptake is important for autophagic flux, the possibility remained that SNX25 loss could disturb other processes, further affecting autophagy. We thus tested if the SNX25 TM or PX domains are required for autophagic flux. Our data demonstrated that, autophagic flux is rescued by expressing either SNX25 PX- or TM-deleted mutants in *SNX25* KO cells. These results indicated that a SNX25 TM mutant unable to rescue VAMP8 endocytosis can still rescue the autophagy defects caused by *SNX25* deletion. Thus, additional processes are impaired by *SNX25* loss.

The SNX13, SNX14, SNX19 and SNX25 subfamily has well-known roles in lipid droplet formation and metabolism. Moreover, *SNX14* deletion affects the relative ratio between phosphatidylcholine and phosphatidylserine. Thus, we performed a rescue experiment with ethanolamine (ETA) to test whether increased intracellular phosphatidylcholine concentrations can rescue *SNX25* loss. Using various ETA concentrations, we rescued SNX25 loss of function. These results suggest that SNX25 influences both VAMP8 internalization as well as cellular lipid metabolism (Fig. 1B).

Altogether, our manuscript uncovered a conserved role for *snz* and *SNX25* in the regulation of autophagy through modulation of Vamp7/VAMP8 endocytosis as well as lipid metabolism. Given our ability to rescue *SNX25* loss with different *SNX25* deletion mutants or by ethanolamine supplementation, we posit that distinct *SNX25* domains may partially compensate for *SNX25* loss by modifying specific metabolic or trafficking pathways. Accordingly, reintroducing a partially functioning mutant or modulating specific lipid species abundance rescue *SNX25-*deleted cells. How homeostatic conditions or cellular stressors regulate snz and the SNX25 subfamily, Vamp7/VAMP8 endocytosis, and lipid handling, will be a truly interesting area of research.
